# Worldwide inertia to the use of cardiorenal protective glucose-lowering drugs (SGLT2i and GLP-1 RA) in high-risk patients with type 2 diabetes

**DOI:** 10.1186/s12933-020-01154-w

**Published:** 2020-10-23

**Authors:** Guntram Schernthaner, Naim Shehadeh, Alexander S. Ametov, Anna V. Bazarova, Fahim Ebrahimi, Peter Fasching, Andrej Janež, Péter Kempler, Ilze Konrāde, Nebojša M. Lalić, Boris Mankovsky, Emil Martinka, Dario Rahelić, Cristian Serafinceanu, Jan Škrha, Tsvetalina Tankova, Žydrūnė Visockienė

**Affiliations:** 1 Medical University of Vienna, Vienna, Austria; 2 Institute of Diabetes, Endocrinology and Metabolism, Rambam Health Care Campus and the Bruce Rappaport Faculty of Medicine, Technion, P.O. Box 9602, 3109601 Haifa, Israel; 3 Head of Endocrinology, Russian Medical Academy of Continuous Professional Education, Ministry of Healthcare of the Russian Federation, Moscow, Russia; 4 Department of Internal Medicine #3, Astana Medical University (NpJSC “AMU”), 49A Beybitshilik Street, Nur-Sultan City, Kazakhstan; 5 Division of Endocrinology, Diabetes, and Metabolism, University Hospital Basel, Basel, Switzerland; 6 Division of Gastroenterology, University Center for Gastrointestinal and Liver Diseases, St. Clara Hospital and University Hospital, Basel, Switzerland; 7 5th Medical Department With Endocrinology, Rheumatology and Acute Geriatrics, Vienna Health Association Clinic Ottakring, 37 Montleartstraße, 1160 Vienna, Austria; 8 Department of Endocrinology, Diabetes and Metabolic Diseases, University Medical Center Ljubljana, 7 Zaloška Cesta, 1000 Ljubljana, Slovenia; 9 Department of Internal Medicine and Oncology, Semmelweis University, 2/a Korányi Sándor Utca, Budapest, 1083 Hungary; 10 Riga Stradins University, Riga, Latvia; 11 Riga East Clinical Hospital, Riga, Latvia; 12 Clinic for Endocrinology, Diabetes and Metabolic Diseases, Clinical Center of Serbia, Faculty of Medicine, University of Belgrade, Belgrade, Serbia; 13 Department of Diabetology, National Medical Academy for Postgraduate Education, Kiev, Ukraine; 14 National Institute of Endocrinology and Diabetology, Lubochna, Slovak Republic; 15 Vuk Vrhovac University Clinic for Diabetes, Endocrinology and Metabolic Diseases, Merkur University Hospital, Zagreb, Croatia; 16 School of Medicine, University of Zagreb, Zagreb, Croatia; 17 Faculty of Medicine, Josip Juraj Strossmayer University of Osijek, Osijek, Croatia; 18 Department of Diabetes, Carol Davila University of Medicine and Pharmacy, Bucharest, Romania; 19 Department of Nephrology/Dialysis, N C Paulescu National Institute for Diabetes, Nutrition and Metabolic Diseases, Bucharest, Romania; 20 3rd Department of Internal Medicine, 1st Faculty of Medicine, Charles University, 1 Ulice Nemocnice, 128 08 Prague 2, Czech Republic; 21 Department of Endocrinology, Medical University — Sofia, 2 Zdrave Street, Sofia, Bulgaria; 22 Clinic of Internal Diseases, Family Medicine and Oncology, Institute of Clinical Medicine, Faculty of Medicine, Vilnius University, Vilnius, Lithuania

**Keywords:** Type 2 diabetes, Cardiorenal protection, Glucose lowering drugs, Clinical inertia

## Abstract

The disclosure of proven cardiorenal benefits with certain antidiabetic agents was supposed to herald a new era in the management of type 2 diabetes (T2D), especially for the many patients with T2D who are at high risk for cardiovascular and renal events. However, as the evidence in favour of various sodium–glucose transporter-2 inhibitor (SGLT2i) and glucagon-like peptide-1 receptor agonists (GLP-1 RA) accumulates, prescriptions of these agents continue to stagnate, even among eligible, at-risk patients. By contrast, dipeptidyl peptidase-4 inhibitors (DPP-4i) DPP-4i remain more widely used than SGLT2i and GLP-1 RA in these patients, despite a similar cost to SGLT2i and a large body of evidence showing no clear benefit on cardiorenal outcomes. We are a group of diabetologists united by a shared concern that clinical inertia is preventing these patients from receiving life-saving treatments, as well as placing them at greater risk of hospitalisation for heart failure and progression of renal disease. We propose a manifesto for change, in order to increase uptake of SGLT2i and GLP-1 RA in appropriate patients as a matter of urgency, especially those who could be readily switched from an agent without proven cardiorenal benefit. Central to our manifesto is a shift from linear treatment algorithms based on HbA1c target setting to parallel, independent considerations of atherosclerotic cardiovascular disease, heart failure and renal risks, in accordance with newly updated guidelines. Finally, we call upon all colleagues to play their part in implementing our manifesto at a local level, ensuring that patients do not pay a heavy price for continued clinical inertia in T2D.

## Introduction

It has been 5 years since we first learned that empagliflozin can save lives and substantially reduce cardiovascular (CV) risk in patients with type 2 diabetes (T2D) and CV disease (CVD) [1]. Subsequently, various cardiovascular and renal benefits have been demonstrated for empagliflozin and other agents in the sodium–glucose transporter-2 inhibitor (SGLT2i) [2–13] and glucagon-like peptide-1 receptor agonist (GLP-1 RA) [10, 14–19] classes. Studies have also now demonstrated these benefits in patients with a more diverse set of diabetic comorbidities, including patients with chronic kidney disease (CKD; the CREDENCE and DAPA-CKD studies [6, 20]) and patients with heart failure (HF) with reduced ejection fraction (HFrEF; the DAPA-HF and EMPEROR-Reduced studies [9, 21]).

In contrast to SGLT2i and GLP-1 RA, numerous CV outcomes trials (CVOTs) have pointed to a generally neutral effect with dipeptidyl peptidase-4 inhibitors (DPP-4i) [22]. However, despite the high prevalence of cardiorenal risk in patients with T2D [23–25], DPP-4i remain more widely used than the similarly priced SGLT2i class in patients with T2D and cardiorenal comorbidities, including CVD, HF and CKD [26–30]. Indeed, the majority of eligible patients still do not receive agents for which protective effects have been proven [26–30], presenting an urgent need to increase uptake of SGLT2i and GLP-1 RA as part of the standard of care for managing cardiorenal risk in patients with T2D [10].

We are a group of diabetologists united by a shared concern that patients with T2D are needlessly dying too young of CV causes, and suffering from avoidable HF hospitalisations (HHF) and progression of CKD. Our strong belief is that clinical inertia is the main driver limiting SGLT2i and GLP-1 RA therapy initiation in eligible patients with cardiorenal risk; importantly, we note that this clinical inertia is contrary to a wealth of evidence from CVOTs, and despite updates to clinical guidelines that encourage SGLT2i and GLP-1 RA use. We also find that low uptake of SGLT2i and GLP-1 RA cannot be primarily explained by pharmacy cost differences, as shown by comparisons with DPP-4i, but instead forms part of a broader picture of clinical inertia in the management of T2D that also affects HbA1c targets.

Here, we summarise clinical inertia as a key barrier to increased use of SGLT2i and GLP-1 RA in eligible, at-risk patients in post-CVOT diabetes care. By doing so, we hope to alert the community to the urgency of the cardiorenal crisis, and the need to do more to mitigate risk in these patients. Plainly, there are many vulnerable patients who could today be switched from other agents to SGLT2i or GLP-1 RA with life-saving consequences, as well as broader benefits for our healthcare systems. In our ‘manifesto for change’, we offer practical suggestions on how to start to dismantle the barriers standing in the way of SGLT2i and GLP-1 RA, and call for all clinicians involved in the care of patients with T2D to play their part in this mission. Central to our manifesto, we propose that the unprecedented results of diabetes CVOTs may necessitate a rethinking of how we view antidiabetic therapy regimens for patients with T2D; in keeping with updated clinical guidelines, we suggest that rather than choosing between prioritising glycaemic control and cardiorenal protection, HbA1c targets and cardiorenal risk considerations should form distinct, parallel treatment algorithms.

We call upon our colleagues to join us in helping to drive a positive change in practice at a local level, as we face a cardiorenal crisis in T2D. By doing so, we can save lives and healthcare resources—preventing some of the thousands of deaths and hospitalisations predicted to be avoidable with increased use of SGLT2i and GLP-1 RA in eligible patients [31].

## Out of step with the evidence: the CVOT-shaped hole in diabetes care

**Fig. 1:**
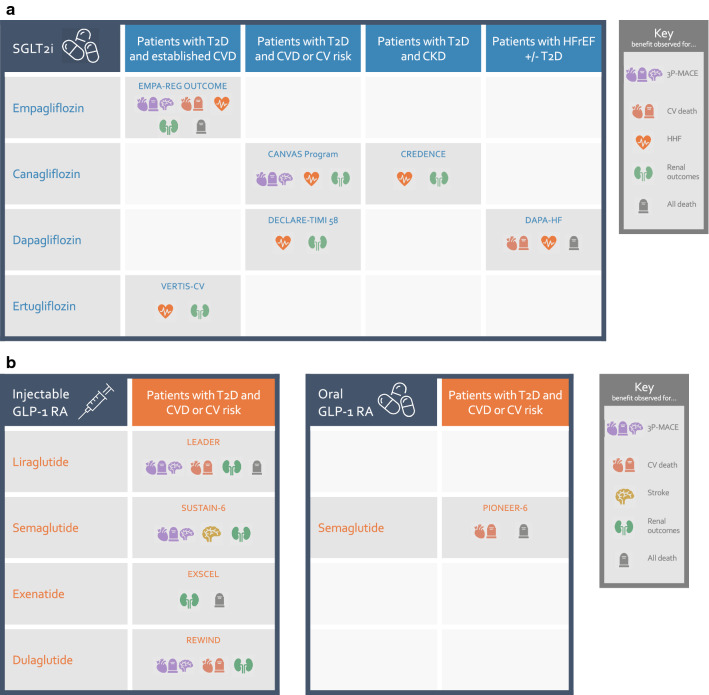
Cardiorenal and mortality benefits reported for SGLT2i and GLP-1 RA. **a** SGLT2i. CVOTs for empagliflozin, canagliflozin, dapagliflozin and ertugliflozin have all pointed to beneficial effects on HHF and renal function outcomes [2–5, 7, 11, 13], either in a population with T2D and established CVD, or in a broader population also including patients with T2D and high CV risk. Renal benefits included slower progression of renal function decline and, where reported, improved albuminuria outcomes. In addition, empagliflozin and canagliflozin CVOTs demonstrated a reduction in 3P-MACE, while empagliflozin alone showed reductions in CV death and death by any cause [1, 7]. Note that in some cases benefits were shown in exploratory analyses. Dedicated renal and heart HF studies are also shedding light on cardiorenal benefits with SGLT2i. In CREDENCE, canagliflozin reduced HHF and renal events in patients with T2D and CKD (and showed a trend towards reduced CV deaths that did reach significance) [6], while in DAPA-HF, CV death, HHF and death by any cause were all reduced with dapagliflozin in patients with HFrEF, with or without T2D [9]. While this manuscript was under review, new publications have also shown that dapagliflozin improved a composite of renal and CV outcomes in patients with CKD, with or without T2D, in DAPA-CKD [20], and that empagliflozin reduced the risk of a composite of CV death or HHF in patients with HFrEF, with or without T2D, in EMPEROR-Reduced [21] (not shown). **b **GLP-1 RA. Some, but not all, GLP-1 RA CVOTs have shown cardiorenal benefits in patients with T2D and established CVD or high CV risk. Among injectable GLP-1 RA, liraglutide, semaglutide and dulaglutide CVOTs have all shown benefits in 3P-MACE and albuminuria outcomes [14, 15, 17, 18, 122, 123]. In addition, liraglutide reduced the risks of CV death and death by any cause, while semaglutide and dulaglutide reduced the risk of stroke. Once-weekly exenatide showed a trend towards a 3P-MACE benefit that did not reach significance in its CVOT, and also suggested reduced risks of death by any cause and albuminuria [19, 124]. Finally, a CVOT on the oral formulation of semaglutide suggested reduced risks of CV death and death by any cause [16]. For all CVOTs, patients in both placebo and treatment arms also received standard of care. Outcome definitions and inclusion criteria varied between CVOTs. Not all outcomes were primary outcomes, and findings may in some cases be of nominal significance only due to multiple testing, e.g. position in a prespecified hierarchy of statistical tests. Only marketed medications are shown

More people with T2D die from CVD than any other cause [32], and the risk of CV death is particularly pronounced for those with renal comorbidities [25, 33, 34]. As death is the ultimate endpoint, mitigating cardiorenal risk is a priority in the management of patients with T2D who are at high CV and/or renal risk [35, 36]. Since 2015, with the publication of the EMPA-REG OUTCOME (empagliflozin) [1] and LEADER (liraglutide) [14] CVOTs, we have had the option of including drugs with proven CV benefits in glucose-lowering treatment regimens [37]. Subsequent disclosures have shown CV benefits for certain additional agents within empagliflozin’s SGLT2i class [4, 7] (Fig. 1a) and liraglutide’s GLP-1 RA class [15–17, 19] (Fig. 1b), as well as suggesting renal benefits across both classes [35, 36, 38].

### What have we learned from CVOTs?

Reviewing all the diabetes CVOT data, we can see that two oral SGLT2i (empagliflozin [1] and canagliflozin [7]) and four injectable GLP-1 RA (liraglutide [14], semaglutide [15], exenatide [19] and dulaglutide [17]) have been proven to reduce the risk of 3-point major adverse CV events (3P-MACE; a composite of CV death, myocardial infarction (MI) and stroke), the prespecified primary outcome, in their respective CVOT populations. In addition, secondary and exploratory CV and HF outcomes in CVOTs have suggested that empagliflozin, liraglutide and oral semaglutide reduce the risk of CV death [1, 14, 16]; injectable semaglutide and dulaglutide reduce the risk of nonfatal stroke [15, 17]; and all SGLT2i reduce the risk of HHF [3, 4, 7, 11] (now also confirmed in the dedicated HFrEF studies DAPA-HF and EMPEROR-Reduced, which both included patients with and without T2D [9, 21]).

Other secondary and exploratory outcomes in CVOTs have also suggested additional benefits of SGLT2i and GLP-1 RA that may be of particular relevance to patients with CVD, including slowed progression of albuminuria [39, 40], and reductions in blood pressure and body weight [13, 32]. For SGLT2i, CVOTs and other studies have consistently pointed to strong protection from renal function decline compared with placebo [10, 12, 38].

Unlike SGLT2i and GLP-1 RA, CVOTs for DPP-4i have universally shown no benefit for primary and other ASCVD outcomes (although all agents have demonstrated safety for these outcomes) [22]. For secondary cardiorenal outcomes, DPP-4i CVOTs have indicated generally neutral effects, with possible exceptions of modest albuminuria benefits and increased HHF risks suggested in some, but not all, studies [22].

The remarkable cardiorenal benefits seen with SGLT2i are also reflected in real-world evidence studies, which encompass more diverse patient characteristics and capture the experience of routine clinical practice. In newly presented 3-year interim data from the EMPRISE real-world evidence study, patients were 48% less likely to die during the study period when taking empagliflozin compared with DPP-4i, and 58% less likely to be hospitalised for HF [41]. Compared with GLP-1 RA, patients were 37% less likely to be hospitalised for HF. Similarly, the CVD-REAL studies have shown that a composite of HHF and all-cause death is substantially reduced with the SGLT2i class compared with other glucose-lowering drugs [42, 43] or DPP-4i [44]. Benefits have also been suggested for renal outcomes with SGLT2i in real-world studies, including when compared with other glucose-lowering drugs (CVD REAL 3 [45]) or specifically with DPP-4i (a cohort study of ~ 60,000 new initiators in Denmark, Norway and Sweden [46]).

EMPRISE and the CVD-REAL series have to date cumulatively encompassed more than 400,000 patients treated with SGLT2i, with a broad range of CV risk at baseline. In both EMPRISE and CVD-REAL, a majority of patients did not have established CVD or HF at baseline [41–43], suggesting that SGLT2i provide cardiorenal protection from very early in the progression of CVD.

Real-world data for cardiorenal outcomes with GLP-1 RA are more limited, beyond the aforementioned EMPRISE study. Three-year outcomes from EMPRISE show that MI rates were similar between GLP-1 RA and empagliflozin, but that HHF was more common with GLP-1 RA, while stroke was less common [41]. In a 2014–2018 study of patients with T2D in North-East Italy, 4298 GLP-1 RA initiators were propensity matched to the same number of SGLT2i initiators. The study found that GLP-1 RA were associated with higher rates of 3P-MACE, MI, HHF and hospitalisation for any CV cause than SGLT2i [47]. Evidence for renal outcomes can be found in a cohort study covering 38,731 new users of GLP-1 RA in Sweden, Denmark and Norway, propensity matched to new users of DPP-4i [48]. GLP-1 RA were associated with 27% reductions in both progression to renal replacement therapy and hospitalisation for renal causes [48].

**Fig. 2:**
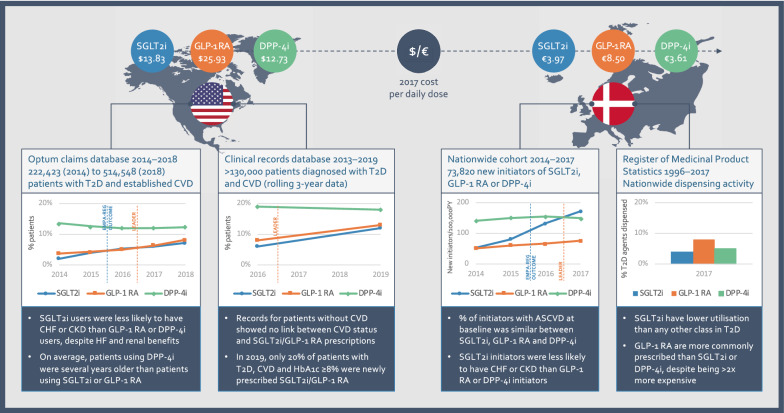
Most patients with T2D and CVD are not prescribed SGLT2i or GLP-1 RA. Data from the US and Denmark show clinical inertia in prescribing SGLT2i or GLP-1 RA, with only modest increases following the disclosures of the first CVOTs to show cardiorenal benefits, in September 2015 (EMPA-REG OUTCOME) and June 2016 (LEADER), respectively. Summaries are shown of data from the US Optum claims database between 2014 and 2018 [26, 31]; a rolling 3-year window study of clinical records from 20 US healthcare organisations, with the oldest cohort from Q1 2013 to Q1 2016 and the most recent cohort from Q1 2016 to 2019 [29, 30]; a nationwide cohort of new initiators of T2D therapies in Denmark from 2014 and 2017 [28]; and a nationwide registry of medicine utilisation in Denmark from 1996 to 2017, which did not include patient-level data [27]. Contemporary costs of SGLT2i, GLP-1 RA and DPP-4i in 2017, the most recent year captured by all the studies, show that pricing does not seem to explain therapy preferences. US prices are median National Average Drug Acquisition Cost reference data per day for empagliflozin, liraglutide and sitagliptin [73]. Other agents in each class were similarly priced. For Denmark, mean prices for a defined daily dose are shown across each class [27]

The evidence from CVOTs—and supporting, confirmatory data from real-world evidence studies—has led guidelines to advocate increasing the use of SGLT2i and GLP-1 RA [35, 36, 49–51] and, from the authors’ perspective, profoundly changed our clinical practice. However, prescribing evidence from North America and Europe suggests that most eligible patients are still not receiving these agents [26–30] (Fig. 2), despite their proven cardiorenal benefits and the urgency of addressing CV and renal risk as comorbidities that pose the biggest threat to life in T2D.

### Why do some SGLT2i and GLP-1 RA show cardiorenal benefits in diabetes CVOTs?

The mechanisms underlying cardiorenal benefits with SGLT2i and GLP-1 RA in patients with T2D are not yet certain, although several have been proposed.

For SGLT2i, suggested mechanisms include (but are not limited to): reduced levels of plasma plasminogen activator inhibitor-1 (PAI-1), leading to visceral fat area loss and a restored adipokine balance [52]; beneficial vascular effects via anti-inflammatory mechanisms, as indicated by reduced high-sensitivity c-reactive protein (hs-CRP) [53]; energy repletion and reduced inflammation mediated by AMPK, together with reduced autophagy and lower levels of CD36 and cardiotoxic lipids, in the heart [54, 55]; haemodynamic volume effects [56]; improved cardiac remodelling, increased provascular progenitor cells and decreased ischaemia/reperfusion injury [57]; and off-target inhibition of the cardiac Na^+^/H^+^ exchanger [58].

Proposed mechanisms for GLP-1 RA include an anti-atherothrombotic effect, as well as amelioration of inflammatory markers, resulting in the enhanced retardation of atherosclerosis [59, 60], together with an antihypertensive effect [61]. This may occur via antiproliferative actions on vascular smooth muscle cells and endothelial cells, reductions in oxidative stress, and increases in nitric oxide generation, microvascular recruitment and microvascular blood flow [61].

### An urgent need to save more lives with SGLT2i and GLP-1 RA

According to recent surveys of prescribing habits, only modest increases in SGLT2i and GLP-1 RA use have been observed during the CVOT era; to compound the issue, prescribing rates for the two classes, as new agents, have historically been low [26–29]. For example, among several hundred thousand US patients with T2D and established CVD who received glucose-lowering drugs between 2014 and 2018, the proportion taking SGLT2i did increase after the publication of EMPA-REG OUTCOME, but only from 4.1% in 2015 to 7.2% in 2018 [26]. Similarly, GLP-1 RA use increased after the publication of LEADER, but only from 4.2 to 8.2% [26]. Thus, the majority of patients with T2D and established CVD in the US were not given a glucose-lowering drug with proven CV benefits as many as 3 years after the cardioprotective effects of empagliflozin became known. It is particularly unfortunate that the historical use of SGLT2i was particularly low in patients with HF [62], who may stand to benefit most from the reduction in HHF observed in CVOTs. For patients with T2D, HF is a common comorbidity with a poor prognosis, and a major cause of mortality [63].

Uptake of SGLT2i and GLP-1 RA may be even lower for patients with CV comorbidities in Europe than in the US, according to national registry data from Denmark. Across all patients with T2D, the registry data show that SGLT2i had only a 4% share of all antidiabetic agents by 2017, with GLP-1 RA faring little better, at 8% [27]. Among new initiators of these agents between 2014 and 2017, only 28–29% had established atherosclerotic CVD (ASCVD) [28]. As this is a similar prevalence to the estimated 20–25% of patients with early T2D in Denmark who have ASCVD, it seems that therapy choices were not optimally targeted to the patients best supported by evidence from CVOTs [28].

By contrast, despite accumulating CVOT evidence that DPP-4i do not provide CV protection, US prescribers maintained a preference for DPP-4i vs SGLT2i in patients with T2D and established CVD [26, 29, 30] (Fig. 2). Indeed, parallel to the emergence of such data, the proportion of patients receiving DPP-4i remained stable from 2015 to 2018, at 12% [26]. Consistent with this finding, a 2014–2017 study focusing on cardiology centres found that patients with established ASCVD or at high risk of CV events were around threefold more likely to be taking DPP-4i than SGLT2i or GLP-1 RA [31, 37]; while a study focusing on primary care from January 2013 to April 2016 found that 23% of >250,000  US patients with T2D received DPP-4i second-line to metformin, compared with 4% receiving SGLT2i and 6% receiving GLP-1 RA [64]. Even by 2019, patients with T2D and CVD were no more likely to be taking SGLT2i or GLP-1 RA than patients without CVD [30].

In Denmark, DPP-4i accounted for 5% of antidiabetic agents given to patients in 2017 [27], with the share of new initiators remaining stable from 2014 [28]. Although new initiators of SGLT2i sharply increased following EMPA-REG OUTCOME, the ratio of SGLT2i:DPP-4i new initiators between 2014 and 2017 was similar regardless of whether patients had ASCVD [28]. This suggests that even where SGLT2i share is increasing, cardioprotective benefits are not fully appreciated by prescribers [28]. Instead, obesity seemed to be a much stronger driver of SGLT2i preference over DPP-4i [28].

We were even more troubled by the usage rates of SGLT2i in a population that may have most to gain, namely patients with chronic HF (CHF). Fewer US patients with T2D, established CVD and CHF were taking SGLT2i than GLP-1 RA or DPP-4i in 2018 [26], even though updated ADA guidelines for that year singled out SGLT2i as reducing the risks of HHF in CVOTs [65]. Among US patients fitting the eligibility criteria for the EMPA-REG OUTCOME trial, the presence of HF actually reduced the likelihood that a patient would be given SGLT2i [31]. Similarly, among new initiators in Denmark between 2014 and 2017, patients with CHF were more likely to be prescribed DPP-4i than SGLT2i [28]. According to a Scandinavian cohort study, the risk of hospitalisation for HF is robustly associated with T2D, even when patients achieve targets for HbA1c, low-density lipoprotein cholesterol, albuminuria, smoking and BP [23].

Another at-risk population that causes us particular concern is the older patient group. Although incidence rates of HHF and mortality are both substantially higher in older than younger patients [41, 66], prescription data show that older age is associated with DPP-4i use in preference to SGLT2i [26, 28, 31]. This may be due to increased prevalence of renal impairment in older patients (especially those with T2D [67]), as patients with CKD were also less likely to receive SGLT2i than GLP-1 RA or DPP-4i [26, 28], in accordance with prescribing information guidance regarding patients with impaired renal function [68]. However, we note that CVOTs have suggested a strong reduction in the progression of CKD with SGLT2i [2, 5, 8], subsequently proven in renal outcomes studies [6, 20]. Furthermore, both EMPA-REG OUTCOME [69] and EMPRISE [66] have reported cardiorenal benefits with empagliflozin for older patients. For example, a subgroup analysis of EMPA-REG OUTCOME found significantly increased protection from 3P-MACE in elderly (age ≥ 65 years; 45% of the study cohort) vs non-elderly patients [67]. The risk of MACE, HF or mortality in patients with T2D and CKD is also most elevated in older patients, suggesting that these patients are particularly in need of agents that reduce the risk of such events [70, 71]. An individualised approach to treatment is warranted for older patients with T2D, balancing cardiorenal comorbidities alongside health status, life expectancy and hyperglycaemia, while paying careful attention to the risk of hypoglycaemia [67].

What is the impact of the continued reluctance to use SGLT2i or GLP-1 RA in patients with T2D with established CVD or at high CV risk? It has been estimated that if all patients meeting the inclusion criteria of EMPA-REG OUTCOME were to take SGLT2i, an additional 920 deaths, 780 CV deaths and 510 HHF could be avoided per 100,000 patients per year [31]. An even greater number of patients fit the inclusion criteria for other SGLT2i CVOTs, with a recent meta-analysis finding that 50% of patients with T2D across multiple countries fulfil the criteria for the dapagliflozin CVOT DECLARE-TIMI 58 [72]. Treating all LEADER-eligible patients with GLP-1 RA could avoid an estimated 300 MIs and 400 CV deaths per 100,000 patients per year [31]. For older patients, the impact may be even more stark; in the EMPRISE cohort, patients ≥ 66 years taking SGLT2i had 757 fewer deaths and 996 fewer HHF per 100,000 patient-years, compared with propensity matched patients taking DPP-4i [66]. We note that less than 50% of these older patients in EMPRISE had a history of CVD at baseline, and only 12% had established HF [66].

## What are the barriers to change?

We believe that all clinicians want what is best for their patients, so it is important to understand the barriers that are preventing SGLT2is and GLP-1 RA from being given to more patients. In our discussions, we identified several possible barriers; importantly, we suggest that clinical inertia rather than cost may be the main driver of continued preference for other agents in these patients.

We concede that the influence of cost will vary according to local reimbursement, but note that pricing for SGLT2i and DPP-4i at the time of the studies described here were similar to one another in the US [73] and Denmark [27] (Fig. 2), and yet prescribers maintained a clear preference for DPP-4i [26–28]. By contrast, clinical inertia is well established as a barrier to treatment intensification in patients with high HbA1c [74–80], and we suggest that this same phenomenon also translates to the management of cardiorenal risk in patients with T2D [81, 82].

### Clinical inertia: the prime suspect

**Fig. 3:**
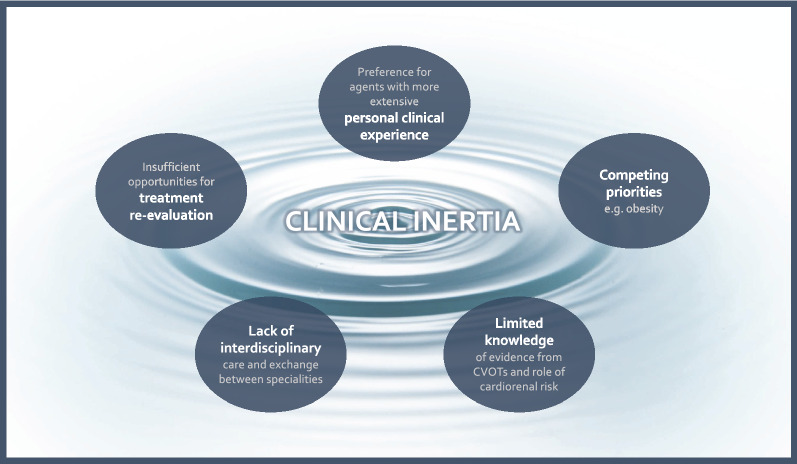
Drivers of clinical inertia in the management of cardiorenal risk in T2D. We argue that the slow uptake of SGLT2i and GLP-1 RA following CVOT disclosures can be attributed to clinical inertia. We suggest several factors that may be responsible for driving this inertia; each will need to be addressed if we are to ensure that eligible, at-risk patients are to benefit from the risk reductions proven in CVOTs, and now emerging from dedicated HF and renal studies

If clinical inertia is the most significant barrier, what could be driving this inertia in the face of the weight of evidence from CVOTs? In its simplest form, inertia is merely an aversion to change; in medicine, this can be well founded, with many healthcare providers understandably preferring to use treatments with which they are more experienced, as they are reassured on the predictability of safety and efficacy, and confident in the practicalities of how to prescribe them. Such tendencies naturally penalise SGLT2i and GLP-1 RA as relatively new treatment classes, despite the evidence favouring their use. However, we believe that lack of awareness, competing priorities, and siloed specialities may all also have important roles in driving clinical inertia in T2D (Fig. 3).

It is our view that not all colleagues are fully aware of the cardiorenal benefits shown in some CVOTs, nor of the central role of CV and renal risk in outcomes for patients with T2D; this may be especially true of primary care practitioners (PCPs), who necessarily have fewer opportunities for education on CVD, renal disease and diabetes than specialists, and may have limited knowledge on evidence for treatment outcomes [83]. Indeed, clinical inertia in glycaemic control has been shown to be more pronounced in PCPs than specialists [77], and glucose levels as well as CV risk factors have been shown to be better controlled with earlier referral to specialist care [84]. That said, we note that clinical inertia on glucose control nevertheless remains quite substantial even among specialists [77], and this may also be the case for cardiorenal risk management, especially given a historical pattern of insufficient attention to CV risk in pre-CVOT diabetes guidelines [85].

We have seen that the opportunities for interdisciplinary collaboration are limited in many settings, which means that PCPs and endocrinologists may not benefit from the relevant knowledge of cardiologists and renal specialists. Related to this, CVD [24] and CKD [86, 87] may be underdiagnosed in patients with T2D. Involving multiple disciplines has been shown to produce desirable outcomes in weight management [88] and glycaemic control [84] in T2D; and we believe that a multidisciplinary approach can be similarly beneficial in the management of cardiorenal risk.

To compound insufficient education on CVOTs, physicians face competing priorities from more apparent risks such as hypoglycaemia and obesity [51]. Indeed, we have observed that obesity is a bigger driver of treatment choice than CV or renal risk when comparing SGLT2i, GLP-1 RA and DPP-4i [28]. Obesity is very obvious to both clinicians and patients; the challenge is to ensure that the less visual risks of CVD, HF and renal disease [24, 89] have sufficient prominence in treatment considerations, given their contributions to mortality in T2D, and CVOT evidence on risk mitigation. The challenge of competing comorbidities is further complicated by the need for physicians to be aware of the interconnected risks within the cardiorenal–metabolic axis, such as the elevated risk of MACE and HF in patients with T2D and CKD [70, 90].

For many patients, their treatment journey within the healthcare system precludes the possibility of regular treatment re-evaluations, even if their level of cardiorenal risk is increasing. Delay in treatment intensification is well characterised for managing hyperglycaemia [79]. Both CV [24] and renal disease [2] are known to progress as duration of T2D increases, but infrequent treatment re-evaluations may mean that there is a lack of opportunity to reassess whether the addition of an agent with proven CV or renal benefits is warranted due to new onset or progression of cardiorenal comorbidities.

### Balancing risks and benefits—who is ‘the right patient’?

One driver of clinical inertia may be the perceived balance between cardiorenal benefit and risk due to the safety profile or potential for undertreatment. In particular, for SGLT2i, a consistent signal has been seen for urogenital infections and rare but potentially serious events of diabetic ketoacidosis (DKA), with especially worrisome cases that have atypical presentation (i.e. non-elevated glucose levels) and so may not be readily detected [91].

It is entirely proper to be cautious about these aspects of SGLT2i, but we believe that the risks are outweighed by the considerable cardiorenal benefits of these agents. Although relatively common, urogenital infections observed in patients on SGLT2i are typically mild-to-moderate and clinically manageable [91]. DKA events with SGLT2i are rare, but should be recognised as potentially very serious [92, 93]. Due to the elevated risk of DKA with SGLT2i in patients with type 1 diabetes [92, 93], clinicians should be alert to the possibility of latent autoimmune diabetes in patients presumed to have T2D. For patients with bona fide T2D, the risks of DKA are lower and can be mitigated by interrupting use for surgery, during prolonged periods of starvation, or in the setting of recurrent illness [92, 93]. Nevertheless, clinicians should be fully appraised of DKA guidance in SGLT2i prescribing information, including clinical settings where monitoring is recommended. So long as these risks are mitigated and monitored, we believe that the safety profiles of SGLT2i should be viewed as broadly favourable, and certainly not of sufficient concern to justify depriving eligible patients of the cardiorenal protective effects of these agents.

Reluctance to use SGLT2i in patients with impaired renal function may often be due to prescribing restrictions [68], in which case GLP-1 RA are preferred for patients with ASCVD or renal risk [36, 51]. In other cases, physicians may nevertheless be concerned that hyperglycaemic efficacy is reduced with SGLT2i in patients with impaired renal function, even if the estimated glomerular filtration rate (eGFR) remains within the permitted prescribing range [94]. However, this overlooks the benefits of SGLT2i for cardiorenal outcomes, which are independent of glycaemic efficacy and seen even in patients with CKD [6, 38]. Furthermore, we do not believe that physicians are compelled to choose between undertreatment for hyperglycaemia and undertreatment for cardiorenal risk; instead, SGLT2i should be maintained while adding a further agent to better control HbA1c levels, as recommended by current guidelines [36, 51].

Beyond patients with diagnosed CKD, the overall low level of use of SGLT2i and GLP-1 RA in patients with T2D suggests that many who have undiagnosed CKD may also not be receiving optimal care. A recent retrospective study of 136,157 patients with T2D in the US between 2011 and 2019 found that more than half of patients with micro- or macroalbuminuria were not diagnosed with CKD, a proportion that did not meaningfully improve across the study period [86]. Similarly, while awareness among physicians of CKD in patients with renal impairment showed a modest improvement over the decade, 40% of patients with eGFR 45–59 ml/min/1.73 m^2^ were still not diagnosed with CKD by 2016–2019 [86]; even for patients with the more severe renal impairment of eGFR 30–44 ml/min/1.73m^2^, 15% remained undiagnosed with CKD, despite these laboratory values being present in their electronic medical records [86]. Diagnosis rates in primary care may be even lower; in a retrospective observational study of 9307 patients with T2D in primary care in the US between 2011 and 2012, only 12% of patients meeting the criteria set out in national guidelines were diagnosed with CKD [87]. For patients with moderate-to-severe (Stage 3–5) CKD, the rate of diagnosis was higher, but still only 22% [87]. Therefore, identifying ‘the right patient’ for treatment with SGLT2i or GLP-1 RA will need to involve improved diagnosis of CKD in order to provide renal protection to the broadest set of eligible patients.

### Does saving lives cost more money?

We accept that reimbursement barriers are highly variable between and within countries, and that in some cases the challenge of reimbursement may tie clinicians’ hands, preventing some patients from accessing the optimal drug for their clinical profile [95, 96]. Where reimbursement does not favour SGLT2i and GLP-1 RA, there may be a gap in reimbursement authorities sufficiently acknowledging the relevance of cardiorenal risk to outcomes in choosing antidiabetic agents.

**Fig. 4:**
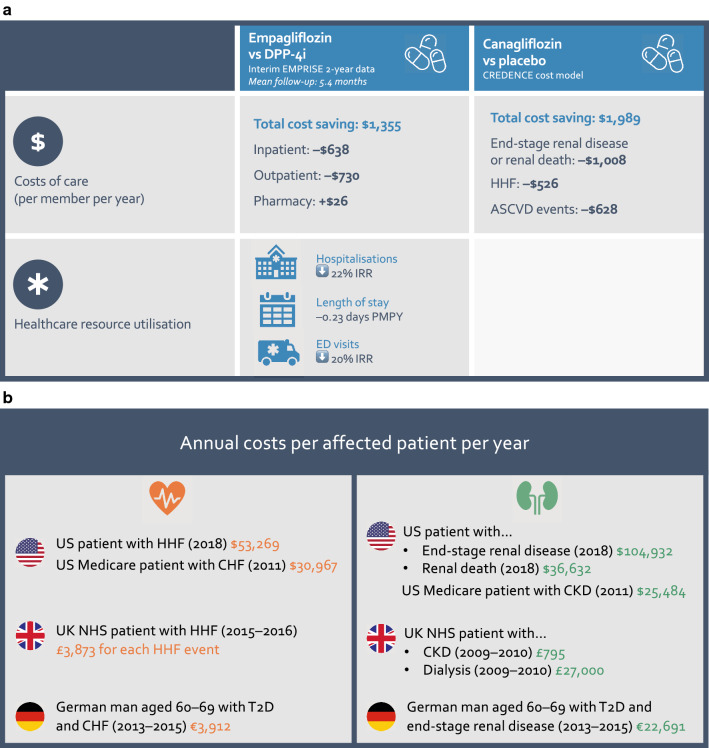
Savings to healthcare costs and resource utilisation with SGLT2i. **a** Savings across US insured populations with T2D. Two-year interim data from the EMPRISE real-world evidence study has measured the cost of care and healthcare resource utilisation for new initiators of empagliflozin vs DPP-4i in two commercial claims databases plus Medicare patients between August 2014 and September 2016, with an average of 5.4 months follow-up. Healthcare resource utilisation data were available for 17,549 patients in each arm matched 1:1 by propensity scoring, and showed ≥ 20% reductions in the numbers of hospitalisations and ER visits with empagliflozin per member per year (PMPY) [125]. Cost data were available for 2928 patients in each arm matched 1:1 by propensity scoring, and showed substantial savings with empagliflozin across the full cohort [126]. A model based on data from the CREDENCE renal outcomes study estimated that the total cost saving for a US insured population with T2D and CKD would be nearly $2000 PMPY when adding canagliflozin to standard of care [100]. **b **Savings per affected patient in the US, UK and Germany. Cost data from the US [100, 103], UK [101, 127] and Germany [102] showing the healthcare expenditure associated with HF and CKD. As expected, costs for the US are notably higher than in Europe; however, even in the UK and Germany expenditure is substantial

Indeed, the full cost of T2D to the healthcare system encompasses considerable healthcare resource utilisation and expenditure across the full spectrum of comorbidities, all of which need to be considered when justifying the cost effectiveness of a treatment. For example, increased protection from HF in patients on SGLT2i has been shown to considerably reduce hospitalisation costs in patients with T2D [97–99] (Fig. 4a), while slowed progression of renal disease with SGLT2i can amount to tremendous cost savings, given the large expenses involved in renal care [89, 100, 101]. In the US, the potential average saving in renal care costs in an insured population with diabetic kidney disease has been modelled as > $1000 per member per year [100] (Fig. 4a), consistent with the estimated annual cost burden of each patient with CKD to the UK’s NHS of £795 [101] (Fig. 4b). Estimates for annual costs for each affected patient with CHF range from €3912 for a German man in his 60s with T2D [102] to $31,000 for a Medicare patient in the US [103]; for end-stage renal disease, estimates range from £27,000 for dialysis expenditure alone in the UK to more than $100,000 in the US for the total cost of care [100] (Fig. 4b). For both HF and CKD, the presence of T2D makes care more expensive and resource intensive, and costs and resource utilisation also become higher with disease progression [89, 104]. Therefore, reducing HF hospitalisations and the progression of CKD with SGLT2i has the potential to be highly cost effective, especially in those markets where drug prices are similar between SGLT2i and DPP-4i classes [98, 99, 105].

### Can we keep up with the fast pace of progress?

Non-adherence to guidelines has been dubbed ‘the enemy of evidence-based practice’ [106]. However, the constant release of data from studies evaluating cardiorenal outcomes with SGLT2i and GLP-1 RA can render guidelines out-of-date soon after publication. Conversely, frequent updates to guidelines to keep up with emerging data can be a challenge for clinicians, especially non-specialists, who may struggle to keep abreast of the latest recommendations [107]. Further difficulties can arise when guidelines are issued by different organisations without always being in agreement [108].

In 2018, it was estimated that only 14% of US patients with T2D and CVD benefited from therapy choices rooted in the 2019 ADA guidelines, highlighting the discordance between fast-evolving evidence and slower-to-change practice [29].

## A manifesto to defeat clinical inertia

**Fig. 5:**
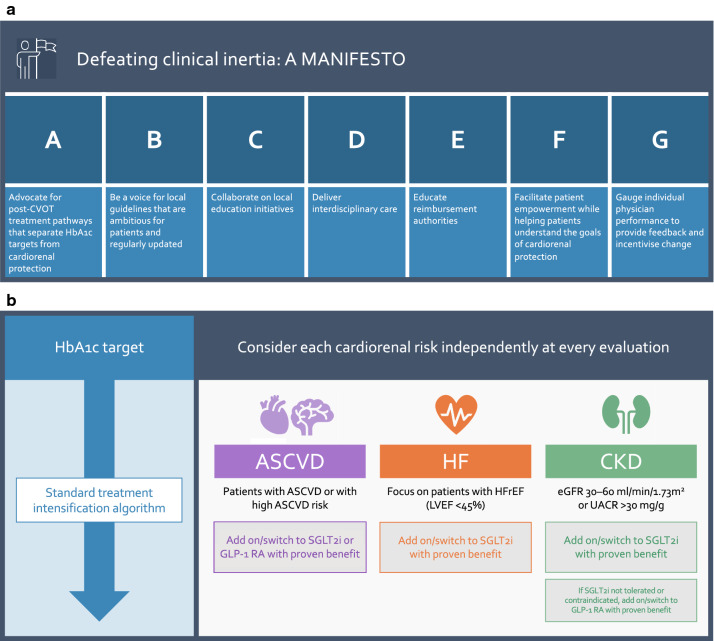
The clinical inertia crisis: a manifesto. **a** Our manifesto for change. We have set out needed actions to tackle clinical inertia in T2D in a ‘manifesto’ to change practice, including seven steps that colleagues can adopt to change local prescribing habits, so that more lives are saved and cardiorenal events avoided with appropriate use of SGLT2i and GLP-1 RA. **b** Rethinking treatment algorithms to separate the management of cardiorenal risk from HbA1c targets. Central to our manifesto is a wider adoption of the new approach taken by ADA, EASD and ESC guidelines [36, 50, 51] to treatment algorithms of antidiabetic agents. Rather than only add on treatments when HbA1c targets are exceeded, updated guidelines recommend that ASCVD, HF and renal risks should be considered independently of HbA1c. For patients with established ASCVD or presenting indicators of high ASCVD risk, the treatment regimen should be adapted by add-on or switch to incorporate a SGLT2i or GLP-1 RA with proven CV benefits. For patients with HF, notably HFrEF with LVEF < 45%, or CKD, an SGLT2i with proven benefit should be incorporated by add-on or switch, so long as not contraindicated e.g. due to renal function lower than indicated in prescribing information. CKD can be defined as estimated glomerular filtration rate (eGFR) < 60 ml/min/1.73 m^2^ and/or urinary albumin–creatinine ratio (UACR) > 30 mg/g (and especially if > 300 mg/g). Among SGLT2i, according to current European Union prescribing information, dapagliflozin, empagliflozin and ertugliflozin should not be initiated below 60 ml/min/1.73 m^2^ (and stopped if persistently below 45 ml/min/1.73 m^2^), while canagliflozin should only be used below 45 ml/min/1.73 m^2^ for patients with UACR ˃ 300 mg/g, and should not be initiated below 30 ml/min/1.73 m^2^. Prescribing information in the United States has some differences; ertugliflozin is not recommended in patients with an eGFR below 60 ml/min/1.73 m^2^, while canagliflozin, dapagliflozin and empagliflozin may be initiated below 60 ml/min/1.73 m^2^, but should be discontinued if persistently below 45 ml/min/1.73 m^2^ (with the exception of dapagliflozin in patients with HFrEF, with or without T2D, where use is supported for eGFR ≥ 30 ml/min/1.73 m^2^). If SGLT2i cannot be used, a GLP-1 RA with proven benefit should be considered to improve renal outcomes for patients with CKD

We believe that change is urgently needed to reduce clinical inertia and surmount other barriers to using agents with optimal cardiorenal benefits for patients with T2D. We would like to suggest several readily achievable approaches towards this goal, in our ‘manifesto’ to save lives, reduce the occurrence of CV events and HF hospitalisations, and slow the progression of renal disease for our patients (Fig. 5a). Our suggestions are based on our own clinical experience, together with observations reported in the literature discussing clinical inertia more widely in T2D, and our perspective on the latest guideline updates in light of CVOTs.

### Advocate for post-CVOT treatment pathways that separate HbA1c targets from cardiorenal protection

The latest international guidelines have reimagined treatment pathways for glucose-lowering drugs in T2D, separating HbA1c from cardiorenal risk, including distinct considerations for ASCVD, HF and CKD [35, 36, 50, 51] (Fig. 5b). This is a substantial shift from pre-CVOT guidelines, which we believe did not pay sufficient heed to CV risk in patients with T2D [85]. We cannot overstate how crucial we think it is for this multi-faceted, parallel approach to treatment selection to become the standard of care. As the guidelines acknowledge, it is clear from CVOTs that reduced risks of cardiorenal events are independent of glucose control. Therefore, the historical linear approach to treatment intensification based on HbA1c targets is a legacy of the pre-CVOT era, when independent cardiorenal benefits were not known, and should now be consigned to the past.

Learnings from CVOTs also necessitate a rethinking of when to re-evaluate a patient’s treatment regimen. More opportunities will be needed to assess cardiorenal risk, so that missed glucose targets are not the only trigger for re-evaluation. We have a particular concern that long-standing patients may be most vulnerable to clinical inertia and least likely to benefit from updated guidelines. These patients may not have regular treatment evaluation schedules, and treating physicians may be reluctant to change antidiabetic treatments when well-maintained glucose control has been established over a period of time, irrespective of the cardiorenal risks presented. As cardiorenal disease is expected to progress over time with duration of T2D, it is essential that treatment for these patients is re-evaluated to consider prescribing SGLT2i or GLP-1 RA according to the balance of clinical risk factors for ASCVD, HF and CKD.

For new patients, early selection of an agent with cardiorenal benefits will reduce the requirement for later evaluations. Guidelines recommend that cardiorenal risk is considered from first add-on to metformin [36, 51] (or, in recommendations from the European Society of Cardiology (ESC), from first diagnosis of T2D, although this has been controversial and for some patients would translate to unlicensed SGLT2i or GLP-1 RA monotherapy [50, 108]). Early in the course of T2D, satisfaction with HbA1c levels may mean that physicians are less likely to add on an SGLT2i or GLP-1 RA, but we note that a substantial burden of CVD is present in patients with T2D and HbA1c < 7.5% [32]. For example, a nationwide diabetes register in Scotland showed that 66% of patients with T2D on metformin monotherapy were at very high CV risk [109].

For patients who have already intensified their treatment beyond metformin monotherapy, managing cardiorenal risk may involve a switch from a neutral drug to one with proven protection, thereby reducing the burden of polypharmacy. Switching from other agents to SGLT2i or GLP-1 RA will be a key indicator of the success of our manifesto. Although we recognise the physician’s natural intuition not to change medication in a patient with well-controlled Hb1Ac, for the many patients with on-target HbA1c where CV risk, HF or CKD is a factor [32], it may be that poorly controlled cardiorenal disease is being missed. For this reason, we believe that treatment algorithms should very clearly encourage such patients to be switched as a matter of urgency to SGLT2i or GLP-1 RA.

### Be a voice for local guidelines that are ambitious for patients and regularly updated

As we have seen with the rapid emergence of CVOT data, without regular updates guidelines can soon become out of step with the latest evidence. While we have discussed the challenge of international guidelines keeping apace of new CVOT data, committees developing local guidelines may have limited resources that make this an even more acute problem. Furthermore, clinicians — in particular PCPs — may be overburdened with numerous guideline updates calling on their attention; as such, the benefit from an evidence-based medicine standpoint of regularly producing updates may be countered by clinical apathy to yet-another-guideline, thus limiting effective communication.

Nevertheless, we believe that up-to-date, well-communicated guidelines are at the core of any successful strategy to overcome clinical inertia. Therefore, it is incumbent upon us to be advocates for local guidelines that have simplified, clear and practical recommendations for busy clinicians to easily digest. It is crucial that these guidelines are centred on a key message of the need for a change in prescribing habits—robustly encouraging treating physicians to use newer drug classes even where personal experience is limited, and conveying the urgency for eligible patients to receive agents with proven cardiorenal benefits, either by switching or as add-on therapies.

We also suggest putting in place a mechanism for triggering frequent guidelines updates, if not already present, to ensure that the latest evidence is accounted for.

### Collaborate on local education initiatives

We have acknowledged the lack of awareness that may be a key driver of clinical inertia. We can all have a role in creating and supporting the delivery of local education initiatives, especially for PCPs, to ensure that the cardiorenal benefits of SGLT2is and GLP-1 RAs are more widely understood and appreciated. This should go alongside education on the central and interconnected roles of CV and renal risk in outcomes for patients with T2D, to persuade both PCPs and specialists that these risks demand more attention and need to be addressed independently of glycaemic control and obesity.

### Deliver interdisciplinary care

By providing more opportunities for interdisciplinary collaboration, we can increase exchange of expertise and ensure appropriate specialist attention earlier in the course of the disease. Although we note that cross-speciality exchange on its own may not be sufficient to improve CV risk management [82], early involvement of a multidisciplinary team in T2D care has been shown to reap clinical benefits [110]. We agree with a recent call to cardiologists to become more involved in supporting diabetology and primary care colleagues in the management of CV risk in patients with T2D [37]. As more data emerge on renal benefits with SGLT2i, we suggest that similar involvement from nephrologists will be required. A greater focus on interdisciplinary care will also better serve the growing understanding of T2D as part of a cardiorenal–metabolic axis, with prevalent cardiorenal and metabolic comorbidities that each carry an incremental risk of MACE, HF and mortality [70, 71, 90].

### Educate reimbursement authorities

Cost is evidently not the main barrier to uptake of SGLT2i and GLP-1 RA, as we have discussed; however, we recognise that for some healthcare providers it may be the deciding factor, and for others there may simply be no option to prescribe these agents, where local reimbursement rules do not support them. It is our view that clinicians can have an important role in dismantling cost barriers through ensuring that local professional guidelines are up-to-date, and engaging with local reimbursement authorities to ensure that they are aware of the full evidence base supporting these guidelines. This will involve not only communicating the CVOT evidence but also advising on why the management of CV and renal risks are central to outcomes for patients with T2D, as well as decisive in healthcare resource utilisation. For example, a budget impact model in the UK found that treating 194,233 eligible patients with add-on empagliflozin could lead to 2719 avoided heart failure events and 5050 lives saved, with an estimated £19.5 m in cost savings for the NHS [111]. To assess the potential cost and resource savings with SGLT2i and GLP-1 RA, we believe it will be helpful for reimbursement practices to widely adopt the approach taken in international guidelines in separating the clinical requirements for glycaemic control and cardiorenal protection.

### Facilitate patient empowerment while helping patients understand the goals of cardiorenal protection

Currently, too many patients with T2D lack awareness of CV risk factors [112, 113], and even those who are aware may not be optimally improving lifestyle factors to manage risk, or sufficiently accessing specialist care [113]. Similarly, studies suggest that less than 10% of affected patients are aware of the presence of CKD [114, 115], while only a minority of patients are aware that antidiabetic agents are available that can reduce cardiorenal risk [113]. Possible adverse events as set out in the patient information leaflets for SGLT2i and GLP-1 RA may also be more of a concern to patients than the risks of CV events or the progression of renal disease without treatment. The onus is thus on clinicians to ensure that each patient is fully appraised of the balance of risks with and without treatment; by doing so, patients will be empowered to make informed decisions.

Patient education on cardiorenal risk may also have a role in defeating clinical inertia, through empowering patients to discuss their health more fully with their clinicians—and motivating them to adopt lifestyle practices and ask for treatments that will provide cardiorenal protection. Discussions initiated by patients can lead to improved patient recall of treatment information [116], while patient feedback to physicians has been suggested to reduce clinical inertia [75].

What practical steps can we take to support patient empowerment? Encouraging motivated patients to prepare questions before a consultation, and to bring a companion, can increase active participation in decision-making [117]. Outside of the clinic, patient access to digital information can help to reduce the levels of CV risk factors, underscoring the importance of education [118].

### Gauge individual physician performance to provide feedback and incentivise change

Working with clinicians to improve patient outcomes through audit and personal feedback processes may help clinicians to recognise biases or gaps in their knowledge of guidelines [77, 119]. Feedback may involve chart audits, patient surveys or direct observation, and has been suggested to increase physician compliance with recommended care by up to 70% [77]. Indeed, a systematic review of computerised decision support systems for T2D in primary care found that these systems only improved patient outcomes when combined with performance feedback to the PCP [120]. Incentives can also be incorporated into the process as additional motivation, such as institutional accreditation; adding incentives has been shown to be effective in a majority of studies [77]. Financial incentives have also been used with success in T2D, such as in the UK where the NHS introduced a “pay-per-performance” scheme linked to 129 indicators covering different areas including diabetes; this scheme was successful in improving outcomes for the selected indicators, in part due to increased DPP-4i use [121].

To apply these principles to addressing clinical inertia in treating patients with T2D for cardiorenal risk, we suggest that “key performance indexes” are locally implemented for treating physicians. Using electronic records, insurers or other organisations with responsibility for patient outcomes can track each physician’s usage of SGLT2i and GLP-1 RA in eligible, at-risk patients. Targets could be set for increasing the proportion of such patients receiving cardiorenal protective glucose-lowering drugs over time, and physicians could be supported with individual feedback, such as suggesting switching to SGLT2i or GLP-1 RA in appropriate patients, and with computerised decision aids.

## Conclusions

We are very fortunate to be living in the age of the CVOT; while colleagues practising before us had very limited options for improving cardiorenal outcomes, we now have several antidiabetic agents with proven cardiorenal benefits, and in some cases the ability to prolong life. Given this newfound fortune, it is regrettable that many eligible, at-risk patients are still not receiving these agents, and may as a result suffer avoidable disease progression that incurs substantial use of healthcare resources and ultimately premature death. As clinical inertia has remained strong in the face of 5 years of accumulating evidence showing the potential of certain SGLT2i and GLP-1 RA to reduce cardiorenal events in at-risk patients, we are concerned that the status quo will continue. To illustrate the gravity of the situation for patients, we believe that inadequate cardiorenal protection in T2D should now be recognised as a crisis.

As such, we call upon our colleagues to join us in working together across specialities at a local, national and international level to address clinical inertia; ensure guidelines are continuously updated as needed; and support initiatives that will increase access to antidiabetic agents with proven cardiorenal benefit to patients on the basis of cardiorenal risk, and independently of HbA1c goals. Only then will the full promise of CVOT evidence be realised for our patients with T2D.

## Data Availability

All data generated or analysed during this study are included in this published article.

## Abbreviations

ASCVD: Atherosclerotic CVD
CKD: Chronic renal disease
CV: Cardiovascular
CVD: CV disease
CVOT: CV outcomes trial
DKA: Diabetic ketoacidosis
DPP-4i: Dipeptidyl peptidase-4 inhibitors
GLP-1 RA: Glucagon-like peptide-1 receptor agonist
HF: Heart failure
HHF: HF hospitalisation
IRR: Incidence rate ratio
PCP: Primary care practitioner
PMPY: Per member per year
SGLT2i: Sodium–glucose transporter-2 inhibitor
T2D: Type 2 diabetes
